# Impact of dietary intervention on eating behavior after ischemic stroke

**DOI:** 10.3389/fnut.2023.1067755

**Published:** 2023-06-22

**Authors:** Samuel Besseau, Eric Sartori, Pauline Larnier, François Paillard, Bruno Laviolle, Guillaume Mahé

**Affiliations:** ^1^Department of Family Medicine, Faculty of Medicine of Rennes, Rennes, France; ^2^Department of Neurology, Hospital of Lorient, Lorient, France; ^3^Vascular Medicine Unit, CHU Rennes, University Hospital, Rennes, France; ^4^Cardiovascular Prevention Centre, University Hospital, Rennes, France; ^5^Clinical Investigation Center, INSERM, Rennes, France; ^6^Univ Rennes, Rennes, France

**Keywords:** diet, dietary consultation, ischemic stroke, nutrition, questionnaire

## Abstract

**Objective:**

Ischemic stroke is a major health issue. Currently, the relationship between dietary patterns and the occurrence of cardiovascular diseases including stroke is established, but the effect of systematic dietary intervention on dietary changes in ischemic stroke patients is unknown. Our objective was to compare changes in the dietary pattern of ischemic stroke patients who received a systematic diet intervention with changes in the dietary pattern of ischemic stroke patients who did not receive a systematic dietary intervention during their hospitalization.

**Methods:**

In this before-and-after study, two groups of patients with ischemic stroke were compared: Group 1 included 34 patients admitted with an ischemic stroke without a systematic dietray intervention; Group 2 included 34 patients admitted with an ischemic stroke with a systematic dietary intervention. Dietary patterns were assessed by a validated food frequency questionnaire of 19 questions (from a previously validated questionnaire of 14 questions), at the onset of stroke and at 6 months after stroke. This questionnaire allows the calculation of different scores as follows: global food score, saturated fatty acids score (SFA), unsaturated fatty acids score (UFA), fruit and vegetable score, and alcohol score.

**Results:**

Score changes were more important in group 2 than in group 1 for the global food score (7.4 ± 7 vs. 1.9 ± 6.7, *p* = 0.0013), the fruit and vegetable score (2 ± 2.6 vs. 0.6 ± 2.2, *p* = 0.0047), and the UFA score (1.8 ± 2.7 vs. 0.1 ± 3.3, *p* = 0.0238), whereas no significant differences were observed for the SFA score (−3.9 ± 4.9 vs. −1.6 ± 6, *p* = 0.1779) and the alcohol score (−0.4 ± 1.5 vs. −0.3 ± 1.1, *p* = 0.6960).

**Conclusion:**

This study showed that systematic dietary intervention during hospitalization improves the dietary patterns of ischemic stroke patients. The impact on the recurrence of ischemic stroke or cardiovascular events after dietary pattern changes needs to be studied.

## 1. Introduction

Stroke is a major public health issue. Every year, a total of 16 million stroke patients have been hospitalized worldwide (1), while in France, approximately 100,000 patients are hospitalized for stroke and 30,000 for transient ischemic attack (TIA) (2). Among these stroke cases, 87% are ischemic, 10% are hemorrhagic, and 3% are subarachnoid hemorrhage (3). Despite the technical and medical therapeutic advances in recent years, stroke remains a major cause of death and disability in France (2, 4). It is also the second cause of dementia after Alzheimer's disease. The management of these chronic diseases is major health burden in developed countries (5). In France, annual health expenditure for the care of people with stroke is approximately €8 billion. Stroke represents 2% of acute care hospitalizations and 4.5% of rehabilitation care hospitalizations (6). Indeed, 30% of patients are hospitalized in the rehabilitation care units after their stroke.

Many ischemic stroke risk factors have been identified. Among them, non-modifiable factors, such as age (7), sex (8), and family history of stroke (9), and other modifiable factors: high blood pressure (10), smoking (11), diabetes (12), obesity (13), dyslipidemia (14), lack of physical activity (15), chronic alcoholism (16), headache (17), oral contraceptives (18), sleep apnea syndrome (19), chronic kidney disease (20), depression and anxiety (21), and metabolic syndrome, have been found (22).

Among hospitalizations for stroke, a significant proportion concerns recurrent stroke. Recurrences were approximately 20% after 5 years, but variations were observed according to the etiology of the stroke (3). To reduce stroke recurrence, secondary prevention is important. Secondary prevention is important, in particular, in controlling the cardiovascular risk factors and in supporting in the development of lifestyle, dietary rules, and drug therapy.

Eating behavior has an important role in the occurrence of cardiovascular diseases, including ischemic stroke (23, 24). Patients with ischemic stroke have more unfavorable eating habits, more saturated fatty acids (SFA), less unsaturated fatty acids (UFA), and consume more fruits or vegetables compared with the general population (25).

While it is known that the mortality rate of cardiovascular diseases is lower in Mediterranean countries than in northern European countries (26), various studies using validated dietary questionnaires (27) have highlighted that adherence to the Mediterranean diet reduced the mortality caused by cardiovascular diseases (27, 28). Furthermore, no study has shown that the Mediterranean diet reduced the risk of recurrence of ischemic stroke. However, a review of an analysis conducted by the British College of Nutrition and Health mentioned that the presumption of the beneficial effect of this diet is so important that all patients hospitalized for ischemic stroke should receive a dietary intervention (29).

We hypothesized that patients with ischemic stroke had an unfavorable dietary pattern and that a systematic dietary intervention during hospitalization would improve the eating habits of these patients.

The main objective of this study was to compare the eating behavior changes between the onset of ischemic stroke and at 6 months after stroke in two groups of hospitalized patients: Patients in group 1 had a dietary intervention at the discretion of the neurologist (i.e., some had and some did not have dietary counceilling) during the hospitalization, while patients in group 2 had a systematic dietary intervention during hospitalization. Our secondary objective was to compare the modifications of the different food groups and different biomarkers after 6 months in each of the two groups.

## 2. Methods

### 2.1. Type of study

This monocenter before-and-after study was conducted in France in accordance with the French law, all participants gave written informed consent, and the study was approved by the local ethics committee of CHBS (Ref: Avis n°2015/01).

### 2.2. Inclusion and exclusion criteria

All enrolled patients were admitted to the stroke unit of the Neurology Department of Lorient Hospital. The inclusion criteria were patients age of ≥ 18 years old and ischemic stroke defined by a neurological deficit during more than 1 h and confirmed by a neurologist and computerized tomography (CT) or magnetic resonance imaging (MRI) (30).

The exclusion criteria were patients living in institutions or with an active home meal delivery service, patients with cognitive deficit, and patients who could not answer questions by themselves or with the help of a third party.

### 2.3. Study protocol

**Figure 1:**
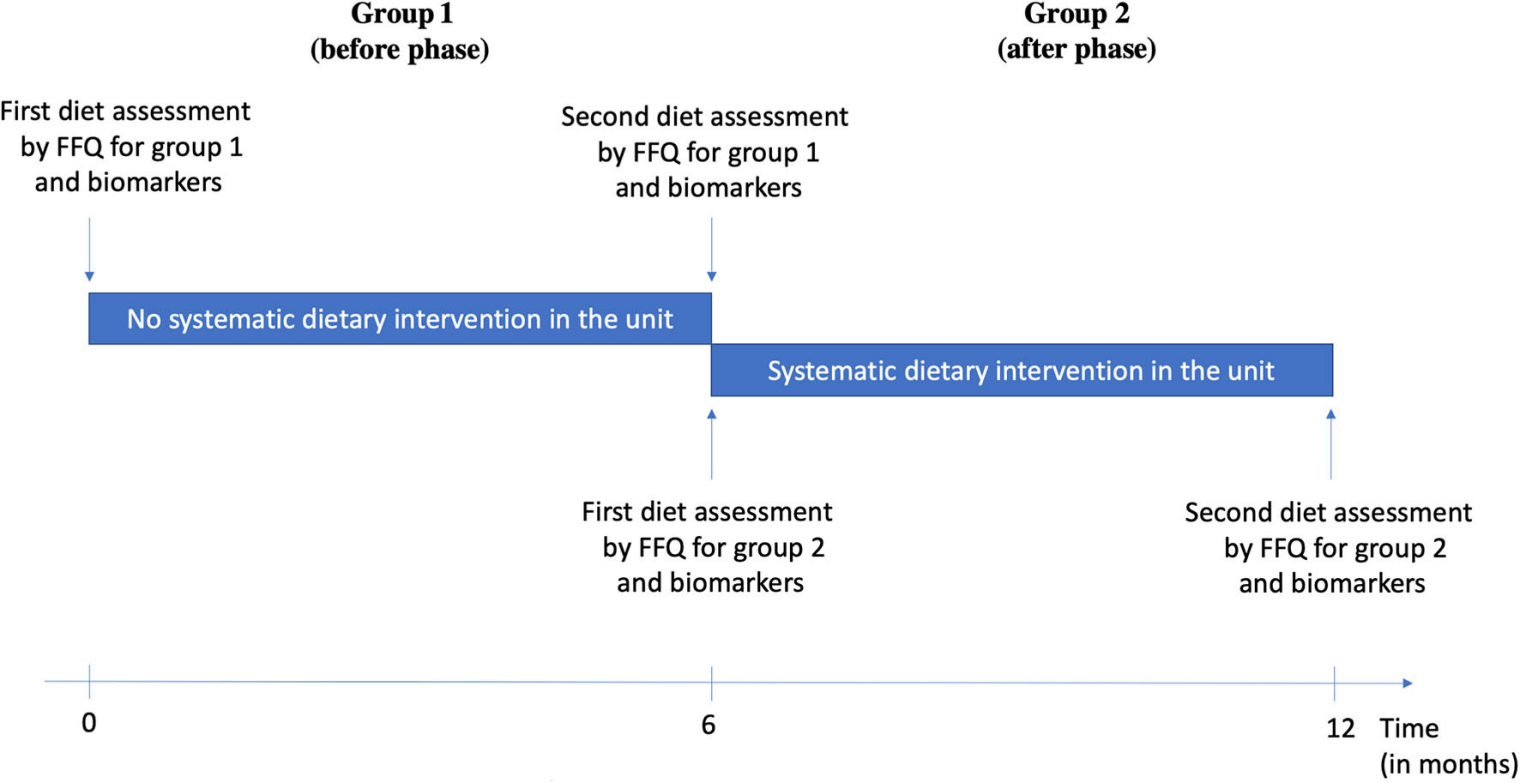
Before-After study design.

Two groups of consecutive patients were included. Group 1 (before phase) included patients who either had or did not have a dietary intervention according to the medical appreciation during the acute phase of stroke (old service protocol). Group 2 (after phase) included patients who had a systematic dietary intervention (new service protocol). Both groups were asked about their eating habits by a questionnaire based on a previously validated questionnaire (31) during the acute phase of ischemic stroke and the same questionnaire 6 months after stroke (Figure 1).

Different classifications have been used to describe patients. The Trial of Org 10172 in Acute Stroke Treatment (TOAST) (32) is a classification according to the etiology of the stroke: TOAST 1: atherosclerosis of large arteries; TOAST 2: embolic heart disease; TOAST 3: lacunar stroke; TOAST 4: other causes; and TOAST 5: undetermined causes (Annex 3). The Barthel Index for Activities of Daily Living measured the inability of 10 activities of daily life (33). This scale ranges from 0 to 100, and the more it decreases, the higher the functional impairment. The modified Rankin Scale (RANKIN; from 0 to 6) assessed disability after stroke (34). The National Institutes of Health Stroke Scale (NIHSS) determined the patients' neurological deficit during the stroke, and the higher the score, the more severe the deficit (35).

For both groups of patients, the following information was collected: age, sex, weight, size, body mass index (BMI), place of residence, professional activity, medical history, high blood pressure, dyslipidemia, coronary heart disease, cardioembolic disease, peripheral artery disease, overweight, smoking, chronic alcohol consumption, and personal history of stroke or TIA, depression, sleep apnea syndrome, thrombophilia, family history of stroke before the age of 45 years or ischemic heart disease before the age of 55 years for the father or before the age of 65 years for the mother, and treatments such as anticoagulants, antiplatelets, anti-hyperlipidemic, lowering blood pressure drugs, antidiabetics, and anti-depressant drugs.

Each patient was measured for inlet blood pressure, glycated hemoglobin (HbA1c), total cholesterol, triglycerides, high-density lipoprotein-cholesterol (HDL-C), and low-density lipoprotein cholesterol (LDL-C).

A semi-quantitative questionnaire of 19 questions from a previously validated one was used to assess eating behaviors with a food score (31). It includes nine questions on SFA: cheese consumption, dairy products, meat, delicatessen, pies or pizzas, pastries, and cooked and raw butter; four questions about fruit and vegetables; four questions on UFA: fish, margarine with omega 3, oil with omega 3, and olive oil; and two questions on alcohol. The SFA score ranges from 0 to −36, UFA from 0 to 29, fruit and vegetables from 0 to 19, and alcohol from 0 to 4. The overall food score ranges from −36 to 47. The higher the score, the more favorable the dietary behavior, and on the contrary, the lower the score, the less favorable the dietary behavior (25, 36). This 19-item questionnaire derived from a 14-item questionnaire was chosen since the 19-item was easier to fill (fewer missing data) and had good reliability as compared with the 14-item questionnaire (31, 37).

Based on the latest recommendations, dietary intervention was conducted by the same trained dietitian of the neurovascular unit where all patients were hospitalized (38).

We measured total cholesterol, HDL-C, triglycerides, LDL-C, and HbA1c in both groups during the acute phase of ischemic stroke and at 6 months after stroke.

### 2.4. Statistical analysis

Sample size calculation: The number of subjects required for the study was 33 patients for each group based on a previous publication to show an improvement of the overall dietary score (25).

Quantitative variables were described as follows: N (number) and mean ± standard deviation. The normality of the distribution of these variables was checked by the Shapiro–Wilk test. When the variables were normal, the groups were compared by parametric Student's *t*-test. Otherwise, the groups were compared by the non-parametric Mann–Whitney Wilcoxon test. For quantitative variables, the number (N) and percentage (%) were presented for each category. Groups were compared using parametric χ^2^ test or non-parametric Fisher's test. For all analyses, a *p* < 0.05 indicated that there was a significant difference.

## 3. Results

**Figure 2:**
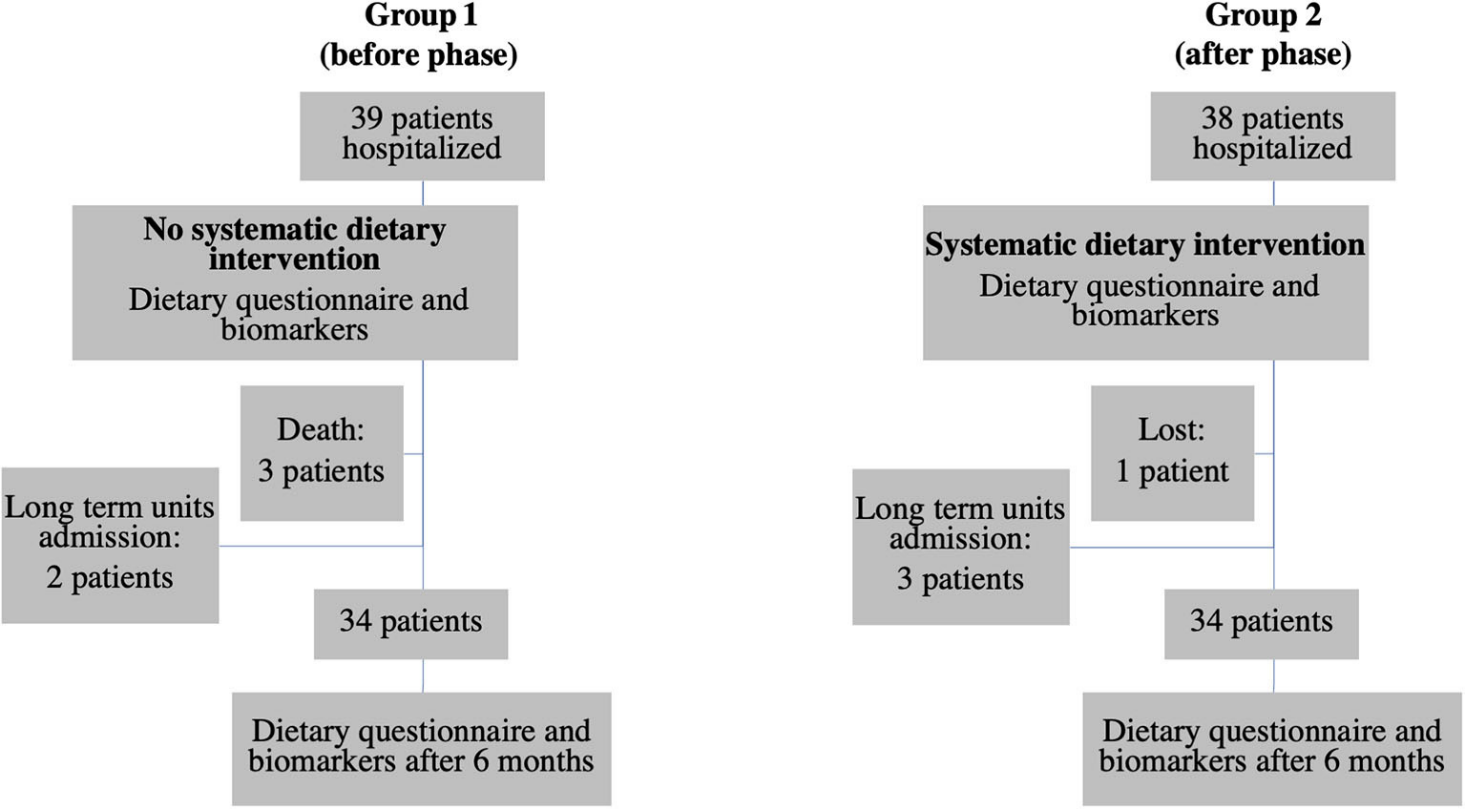
Diagram flow of the patients.

The diagram flow of the patients is presented in Figure 2. In group 1, 39 patients were hospitalized during the study. Three died between 0 and 6 months and two were excluded because they were admitted to a long-term care unit. In group 2, 38 patients were hospitalized during the study. Three were admitted to a long-term care unit and one was lost. In each group, 34 patients were therefore included. Eight patients in the first group and all patients in the second group had a dietary intervention.

The descriptive characteristics of patients in both groups are presented in Table 1. No significant difference was shown between the two groups for the following characteristics: gender, age, weight, height, BMI, place of residence, family situation, professional activity, cardiovascular risk factors, and systolic and diastolic blood pressure at the inlet in the service.

**Table 1:** Description of the population.

	**Group 1: old protocol (*n* = 34)**	**Group 2: new protocol (*n* = 34)**	***p***
Men	26 (76.5%)	20 (58.8%)	*p =* 0.1199 (K)
Age (years)	66.0 ± 13.2	64.9 ± 14.7	*p =* 0.7428 (S)
Weight (kg)	74.9 ± 13.5	75.9 ± 14.9	*p =* 0.7724 (S)
Height (cm)	169.6 ± 9.0	169.1 ± 8.8	*p =* 0.8385 (S)
Body Mass Index (kg/m^2^)	25.9 ± 3.6	26.4 ± 4.4	*p =* 0.6116 (S)
Living area	34	34	*p =* 0.7538 (F)
<2,000 inhabitants	3 (8.8%)	3 (8.8%)	
Between 2,000 and 20,000 inhabitants	24 (70.6%)	21 (61.8%)	
>20,000 inhabitants	7 (20.6%)	10 (29.4%)	
Family situation	34	34	*p =* 0.6368 (F)
Single	4 (11.8%)	2 (5.9%)	
Married	17 (50.0%)	21 (61.8%)	
Divorced	6 (17.6%)	3 (8.8%)	
Widow	3 (8.8%)	5 (14.7%)	
Cohabitation	4 (11.8%)	3 (8.8%)	
Professional activity	34	34	*p =* 0.8156 (F)
Active	10 (29.4%)	9 (26.5%)	
Job seeker	2 (5.9%)	1 (2.9%)	
Sick leave or invalidity	2 (5.9%)	1 (2.9%)	
Retired	20 (58.8%)	23 (67.6%)	
High blood pressure	22 (64.7%)	21 (61.8%)	*p =* 0.8014 (K)
Diabetes	4 (11.8%)	7 (20.6%)	*p =* 0.3232 (K)
Dyslipidemia	18 (52.9%)	16 (47.1%)	*p =* 0.6276 (K)
Ischemic heart disease	4 (11.8%)	1 (2.9%)	*p =* 0.3559 (F)
Cardioembolic disease	6 (17.6%)	6 (17.6%)	*p =* 1.0000 (K)
Peripheral artery disease	2 (5.9%)	1 (2.9%)	*p =* 1.0000 (F)
Overweight	21 (61.8%)	19 (55.9%)	*p =* 0.6222 (K)
Smoking	11 (32.4%)	8 (23.5%)	*p =* 0.4175 (K)
Alcohol consumption	5 (14.7%)	2 (5.9%)	*p =* 0.4275 (F)
History of Stroke or TIA	8 (23.5%)	5 (14.7%)	*p =* 0.3549 (K)
History of anxiety, depression, or sleeping disorders	4 (11.8%)	7 (20.6%)	*p =* 0.3232 (K)
Sleep apnea syndrome	4 (11.8%)	5 (14.7%)	*p =* 1.0000 (F)
Thrombophilia	2 (5.9%)	0	*p =* 0.4925 (F)
Family history of stroke	3 (8.8%)	4 (11.8%)	*p =* 1.0000 (F)
Systolic blood pressure (mmHg)	144 ± 24	147 ± 22	*p =* 0.3143 (W)
Diastolic blood pressure (mmHg)	80 ± 17	76 ± 10	*p =* 0.2956 (W)

Qualitative parameters: number (%), χ^2^ test (K), or Fisher's test (F). Quantitative parameters: mean ± standard deviation, Student's t-test (S), or Mann–Whitney Wilcoxon (W) test. TIA: transient ischemic attack, BMI: body mass index. Group 1 (before phase) included patients who either had or did not have a dietary intervention according to the medical appreciation during the acute phase of stroke (old unit protocol). Group 2 (after phase) included patients who had a systematic dietary intervention (new unit protocol).

The TOAST classification included in group 1: 50% (*n* = 17) TOAST 1, 8.8% (*n* = 3) TOAST 2, 2.9% (*n* = 1) TOAST 3, 0% TOAST 4, and 38.2% (*n* = 13) TOAST 5, while in group 2, we found 55.9% (*n* = 19) TOAST 1, 14.7% (*n* = 5) TOAST 2, 2.9% (*n* = 1) TOAST 3, 0% TOAST 4, and 26.5% (*n* = 9) TOAST 5. There was no significant difference between the two groups (*p* = 0.7469).

The initial RANKIN scores (from 0 to 6) in group 1 were 61.8% (*n* = 21) for RANKIN 0, 17.6% (*n* = 6) for RANKIN 1, and 20.6% (*n* = 7) for RANKIN ≥ 2 and in group 2 were 61.8% (*n* = 21) for RANKIN 0, 23.5% (*n* = 8) for RANKIN 1, and 14.7% for RANKIN ≥ 2 (*p* = 0.7338).

The initial NIHSS was 3.4 ± 3.8 in group 1 and 2.6 ± 1.9 in group 2 (*p* = 0.9402), and the variation in the NIHSS score after 6 months was −2.4 ± 2.8 in group 1 and −1.8 ± 1.8 in group 2 (*p* = 0.4841).

The BARTHEL score was 92.4 ± 17.7 in group 1 and 94.4 ± 11.1 in group 2 at the initial phase (*p* = 0.9320), with a variation of 4.1 ± 12 and 3.5 ± 7.3 (*p* = 0.8462) after 6 months.

The results of the initial food scores in the two groups are presented in Table 2. The food scores for SFA, fruit and vegetables, UFA, alcohol, and global were not significantly different at the initial phase (M0).

**Table 2:** Initial food scores in each group.

	**Group 1: old protocol (*n* = 34)**	**Group 2: new protocol (*n* = 34)**	***p***
SFA	11.4 ± 4.8	10.9 ± 5.5	*p =* 0.7089 (S)
Fruit and vegetables	6.7 ± 2.9	6.4 ± 2.6	*p =* 0.6027 (S)
UFA	8.1 ± 3.7	7.8 ± 4.1	*p =* 0.7813 (S)
Alcohol consumption	1.3 ± 1.7	1.2 ± 1.4	*p =* 0.9735 (W)
Overall score	4.7 ± 7.2	4.4 ± 9.1	*p =* 0.8946 (S)

Quantitative parameters: mean ± standard deviation, Student's t-test (S) or Mann–Whitney Wilcoxon test (W). SFA, saturated fatty acids; UFA, unsaturated fatty acids. Group 1 (before phase) included patients who either had or did not have a dietary intervention according to the medical appreciation during the acute phase of stroke (old unit protocol). Group 2 (after phase) included patients who had a systematic dietary intervention (new unit protocol).

**Figure 3:**
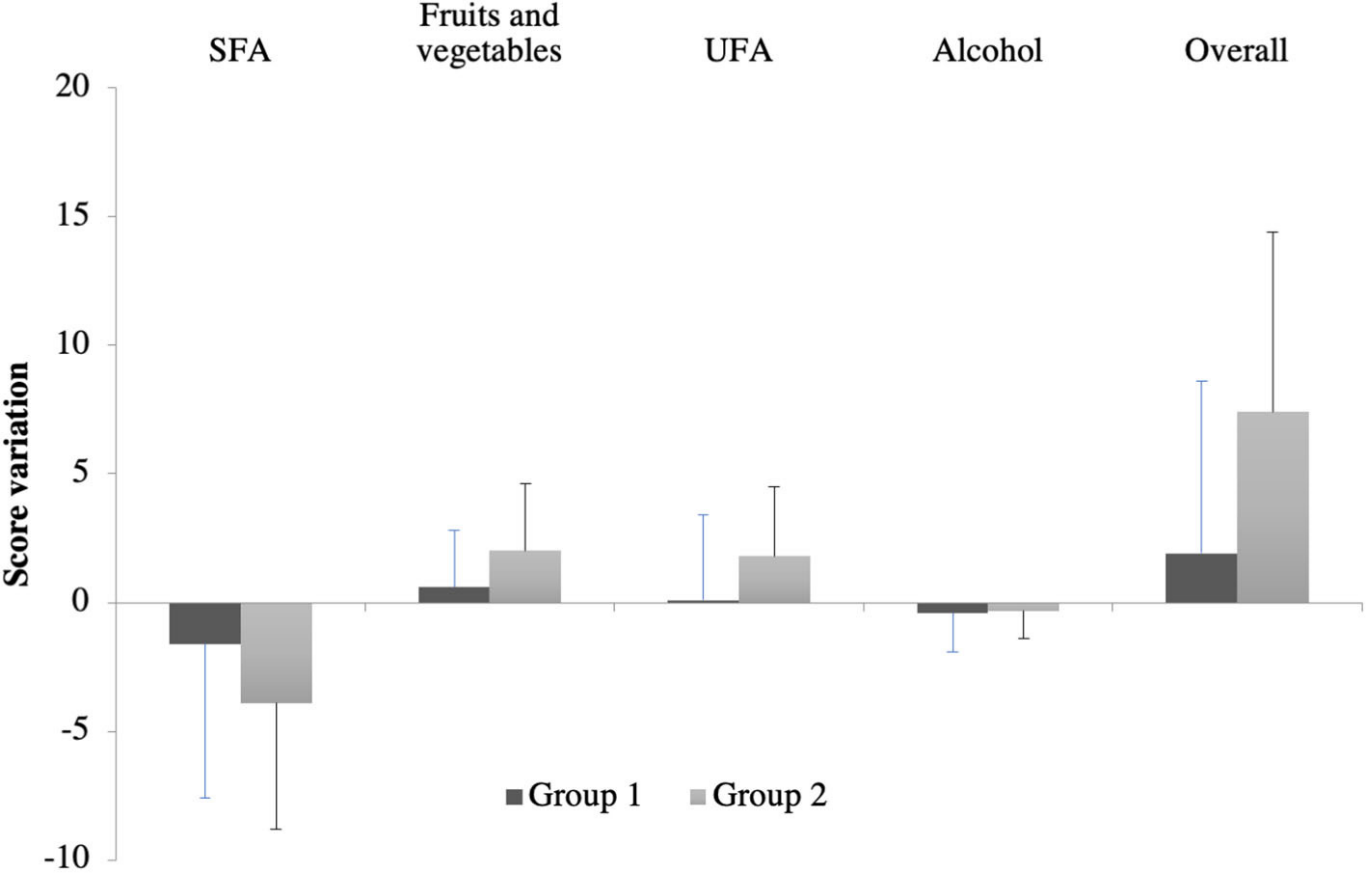
Dietary score variations after 6 months in each group. SFA, saturated fatty acid; UFA, unsaturated fatty acid. Group 1 (before phase) included patients who either had or did not have a dietary intervention according to the medical appreciation during the acute phase of stroke (old unit protocol). Group 2 (after phase) included patients who had a systematic dietary intervention (new unit protocol).

The improvement in the overall score was higher in the group of patients who had had dietary interventions (7.4 ± 7 vs. 1.9 ± 6.7, *p* = 0.0013) (Figure 3). This improvement was also found in the fruit and vegetables score (2.0 ± 2.6 vs. 0.6 ± 2.2, *p* = 0.0047) and UFA score (1.8 ± 2.7 vs. 0.1 ± 3.3, *p* = 0.0238). We did not find significant differences in alcohol score (−0.4 ± 1.5 vs. −0.3 ± 1.1, *p* = 0.6960) and SFA score, although a decreasing trend was observed for the SFA score in group 2 vs. the SFA score in group 1 (3.9 ± 4.9 vs. −1.6 ± 6, *p* = 0.1779).

If taking into account only patients who did not receive a dietary intervention in group 1, it can be observed that the trend to reduce the SFA score was accentuated in group 2 than in group 1 (3.9 ± 4.9 vs. −1.4 ± 5.5), but the difference was not significant (*p* = 0.1685).

After 6 months, there was no significant difference in variations in cholesterol rate and glycated hemoglobin between the groups, despite a higher decrease in the total cholesterol rate (−0.75 ± 1.29 vs. −0.36 ± 1.31 mmol/l) and LDL cholesterol rate (−0.97 ± 1.17 vs. −0.56 ± 1.14 mmol/l) being observed in group 2 more than in group 1 (Table 3).

**Table 3:** Rates and variations in biological markers in each group.

	**Group 1: old protocol**	**Group 2: new protocol**	***p***
Total cholesterol M0 (in mmol/l)	4.59 ± 0.97	5.24 ± 1.07	*p =* 0.0106 (S)
Total cholesterol variation M6 (in mmol/l)	−0.36 ± 1.31	−0.75 ± 1.29	*p =* 0.2457 (S)
HDL cholesterol M0 (in mmol/l)	1.15 ± 0.4	1.23 ± 0.34	*p =* 0.4324 (S)
HDL cholesterol variation M6 (in mmol/l)	0.3 ± 0.4	0.2 ± 0.3	*p =* 0.3278 (S)
LDL cholesterol M0 (in mmol/l)	2.77 ± 0.9	3.41 ± 0.98	*p =* 0.0072 (S)
LDL cholesterol variation M6 (in mmol/l)	−0.56 ± 1.14	−0.97 ± 1.17	*p =* 0.1681 (S)
TG M0 (in mmol/l)	1.48 ± 0.6	1.35 ± 0.54	*p =* 0.4113 (W)
TG variation M6 (in mmol/l)	−0.29 ± 0.58	−0.18 ± 0.4	*p =* 0.3952 (S)
HbA1c M0 (in mmol/mol)	42.64 ± 11.07	40.71 ± 7.51	*p =* 0.1911 (W)
HbA1c variation M6 (in mmol/mol)	−0.84 ± 3.13	−0.58 ± 3.09	*p =* 0.9377 (W)

Quantitative parameters: mean ± standard deviation, Student's t-test (S), or Mann–Whitney Wilcoxon test (W). HDL, high-density lipoprotein; LDL, low-density lipoprotein; TG, triglycerides; HbA1c, glycated hemoglobin. M0, means results at inclusion. M6, means results 6 months after inclusion. Group 1 (before phase) included patients who either had or did not have a dietary intervention according to the medical appreciation during the acute phase of stroke (old unit protocol). Group 2 (after phase) included patients who had a systematic dietary intervention (new unit protocol).

## 4. Discussion

### 4.1. Main results

This study demonstrated that systematic dietary intervention within hospitalization favorably improves dietary habits in patients who had had an ischemic stroke. This dietary improvement was driven by an increase in the consumption of fruit and vegetables and UFA. A decreasing trend in SFA consumption was also observed but was not statistically significant.

Although the importance of the dietary risk factor, including SFA, in the occurrence of ischemic stroke was discussed according to the etiology of stroke (39), adherence to the Mediterranean diet rich in nuts, olive oil, fruits and vegetables, low SFA, dairy products, and red meat is associated with a decreased risk in stroke (26).

The link between SFA and stroke is the most debated (40). Previous studies have demonstrated an association between SFA and stroke (41) but also the opposite (42, 43). SFA are a heterogeneous group of fatty acids that contain only carbon-to-carbon single bonds and differ in their carbon chain length (44). To date, it is recommended that health effects linked to SFA consumption depend on the interacting effects from naturally occurring components and unhealthy compounds introduced by processing (44). For UFA and fruit and vegetables, the link seems clearer (45, 46). In our study, the improvement in the score for fruit and vegetables was more than three times higher in group 2 than in group 1, showing a positive impact of a systematic dietary intervention. A Swedish team has shown that, in primary prevention, consumption of seven fruits or more per day decreased the risk of stroke by 19% compared with those who consumed only one fruit per day (46). Fruit and vegetables that contain vitamins, dietary fibers, polyphenols, and trace minerals may have positive effects on ischemic stroke through several mechanisms: antioxidant activity, decreased platelet aggregation, blood pressure reduction, endothelial function modulation, and anti-inflammatory effects (47–49). Concerning the UFA that contains monounsaturated and polyunsaturated fatty acids, the questionnaire focused on the consumption of fish, margarine, and oil. While with the old protocol (Group 1), there was no improvement in the consumption of UFA, the new protocol (Group 2) has improved the UFA score by 23%. This is of importance since several studies have demonstrated protective effects of polyunsaturated fatty acids consumption on the risk of ischemic stroke (50, 51). Regular consumption of UFA, especially fish, reduces the risk of stroke in primary prevention (45). Of interest, it has been recently shown that the optimal moment to start an intervention to support patients in health-related behavior change after the TIA or ischemic stroke appears directly after the stroke or TIA (52). This strengthens the importance of performing a systematic dietary intervention during hospitalization.

In the primary and secondary prevention of ischemic stroke, the current guidelines recommend a diet low in saturated fatty acids and rich in fruit and vegetables and UFA such as the Dietary Approach to Stopping Hypertension (DASH) diet (53). The DASH diet includes a diet rich in fruit and vegetables and low in salt, sugar, and SFA. It has already shown a decrease in blood pressure (54) and LDL cholesterol levels (55). In our study, significant improvement was not shown in cholesterol levels, despite a decrease of 14.3% (vs. 7.8%) in total cholesterol and 28.4% (vs. 20.2%) in LDL cholesterol in the group with the systematic dietary intervention 6 months after a stroke.

Alcohol consumption was also addressed in the dietary intervention. Heavy drinking is associated with a higher risk of ischemic stroke (56). The systematic dietary intervention for group 2 showed no significant change in alcohol consumption compared with the old protocol (group 1) at 6 months after stroke. However, a decreasing trend in alcohol consumption (less than one drink per day) in both groups at 6 months was observed. Unfortunately, alcohol consumption was only discussed during the dietary intervention when the patient claimed to drink more than three glasses of wine per day for men and more than two glasses of wine per day for women. Based on previous studies, assessment of alcohol consumption should be performed within hospitalization for patients with ischemic stroke (56).

### 4.2. Limitations

Our study has several limitations. First, this study was a monocenter study performed in France with French dietary habits. The results may not be generalizable to other countries. Second, the small number of patients did not permit us to show significant differences in dietary SFA score or biomarkers (total cholesterol and LDL cholesterol), but only improving trends were observed. However, the required number of patients had been calculated on the main objective, which was a change in the overall food score. Third, LDL-C baselines were statistically different between the two groups, and no adjustment was possible due to the number of patients. We cannot exclude that patients in group 2 were more motivated to change their dietary habits than patients in group 1 who had better LDL-c baseline.

## 5. Conclusion

A systematic dietary intervention improves the dietary behavior of patients hospitalized for ischemic stroke by increasing UFA and fruit and vegetable consumption. The effects of dietary improvement (higher fruit and vegetable consumption and higher UFA consumption) after an ischemic stroke remain to be studied using a randomized controlled trial.

## Data availability statement

The raw data supporting the conclusions of this article will be made available by the authors, without undue reservation.

## Ethics statement

The studies involving human participants were reviewed and approved by local Ethical Committee of CHBS (Ref: Avis n°2015/01). The patients/participants provided their written informed consent to participate in this study.

## Author contributions

SB, BL, FP, and GM conceived and designed the analysis. SB and ES collected the data. SB, BL, and GM contributed analysis tools. BL performed the analysis. SB, PL, and GM wrote the paper and which was reviewed by all contributing authors. All authors contributed to the article and approved the submitted version.
